# Evaluation of chromosomal instability in somatic cells of farmed foxes

**DOI:** 10.5194/aab-61-405-2018

**Published:** 2018-10-29

**Authors:** Olga Szeleszczuk, Magdalena Gleindek, Anna Grzesiakowska, Marta Kuchta-Gładysz, Agnieszka Otwinowska-Mindur

**Affiliations:** 1 Department of Animal Anatomy, Institute of Veterinary Sciences, University of Agriculture in Krakow, Krakow 30-059, Poland; 2 Department of Genetics and Animal Breeding, Faculty of Animal Science, University of Agriculture in Krakow, Krakow 30-059, Poland

## Abstract

The micronucleus (MN) test is a common tool used to evaluate cellular genetic instability at the chromosomal level. It determines the effect of physical, chemical and environmental factors on DNA, and thus the body's individual resistance to harmful substances. The karyotypes of blue and silver foxes and their interspecific hybrids are characterized by morphological and structural variation. This variation is partly attributable to the presence of chromosomal polymorphism, which may significantly influence the stability of genetic material in the cells of these species. The objective of the study was to evaluate genetic material stability in selected Canidae species. To this end, analyses using the MN test were performed. Binucleated cells (BNCs) were analysed in microscopic preparations, and the number of micronuclei was determined within these cells. For the proportions of both MN and BNCs, highly significant differences were observed between the fox species. The interspecific hybrids differed from the other fox species in MN percentage. The lowest average was noted in blue foxes (3.33) and the highest in interspecific hybrids (15.21).

## 1 Introduction

The 1970s and 1980s witnessed an increasing number of studies on animals belonging to the Canidae family. Ever since the presence of chromosomal polymorphism was discovered in species such as the arctic fox (*Alopex lagopus*), the common fox (*Vulpes vulpes*) and the Chinese raccoon dog (*Nyctereutes procyonoides),* these animals have been the subject of extensive genetic and biological research. Genome mapping of domestic animals became popular in the early 1990s. As a result, the karyotype of the common ancestor of the order Carnivora was reconstructed as 2n=42 chromosomes (Świtoński et al., 2003). Foxes have been the subject of many studies on account of their productive breeding value. Scientists work towards complete understanding of the effect diet and some feed additives have on body weight gains, behaviour or reproduction in these animals (Przysiecki et al., 2011; Gorajewska et al., 2012; Nowicki et al., 2012, 2013).

The blue fox karyotype has been of interest ever since it became an important breeding animal. The diploid chromosome number in cells of the blue fox is 2n=50, but this karyotype shows a variable number of A chromosomes (2n=48, 49 or 50). This is due to a Robertsonian translocation, as a result of which the number of chromosomes is reduced to 48 or 49 (Mäkinen, 1985a). Robertsonian translocation between two out of four acrocentric chromosomes (pair 23 and 24) is common in cells of the blue fox. It is assumed that the most frequent diploid number of chromosomes is 2n=49, but in terms of health and reproductive parameters, individuals with the 2n=48 karyotype are most prolific (Christensen and Pedersen, 1982; Møller et al., 1985; Świtoński et al., 2006).

Silver fox is a colour variant of the common fox, which has the smallest chromosome number among Canidae species. Initially it was thought that the diploid chromosome number varies from 32 to 42, but in subsequent years this was found to result from the presence of a variable number of B chromosomes (Mäkinen, 1985b; Świtoński et al., 2003). The red fox karyotype was ultimately defined as 2n=34 with a variable number of B chromosomes (0–8; Mäkinen, 1985b; Basheva et al., 2010). All the chromosomes are metacentric except for very small Y chromosome, which morphologically resembles one of the supernumerary chromosomes (Mäkinen and Gustavsson, 1982; Bugno-Poniewierska et al., 2015).

It is common practice in breeding to create interspecific hybrids of both plants and animals (Bugno-Poniewierska et al., 2015). The resulting animals may show increased body weight, better resistance to disease, improved yield and productivity (Short, 1997). The interspecific hybrids of the blue and silver foxes are most often created by crossing female arctic (blue) foxes with male common (silver) foxes. The resultant offspring are viable but infertile, because meiotic chromosome disturbances lead to the formation of abnormal gametes (Mäkinen and Gustavsson, 1982; Bugno-Poniewierska et al., 2015). The number of A chromosomes depends on the presence of a centric fusion in one of the parents (arctic fox), whereas the number of B chromosomes comes from the second parent (common fox) (Mäkinen and Gustavsson, 1982; Bugno-Poniewierska et al., 2015). This may give rise to cellular mosaicism. Mäkinen and Gustavsson (1982) defined the modal number of chromosomes in interspecific hybrids as 2n=41+(1-3) or 2n=42+(1-3). Bugno-Poniewierska et al. (2015) showed that the number of chromosomes in cells varies from 35 to 42. The modal number of A chromosomes was 2n=40, 2n=41 or 2n=42, and that of B chromosomes ranged from 0 to 4 (Bugno-Poniewierska et al., 2015).

Recent years have seen increased interest in research on the impact of various environmental, genetic or lifestyle factors on genetic material stability in humans (Fenech et al., 1999a) but also in plants and animals. Chromosomal instabilities (CIN) are evaluated using cytogenetic tests for chromosome instability such as the micronucleus (MN) test, the sister chromatid exchange test, the bleomycin test, and the comet assay. The main chromosomal instabilities subjected to most analysis are isochromosomes, dicentric chromosomes, micronuclei, sister chromatid exchanges, and fragile sites (Świtoński et al., 2006; Geigl et al., 2008; Kozłowska and Łaczmańska, 2010; Czubaszek et al., 2014; Wójcik et al., 2018).

Micronuclei are small oval-shaped structures found in some eukaryotic cells and localized next to the nucleus of daughter cells after mitotic division. Micronuclei contain acentric and centric chromosome fragments or whole chromosomes (Fenech, 1997). The presence of micronuclei in cells is abnormal. Chromosome loss and non-disjunction are probably caused by spindle or centromere defects, or a decondensed chromosome structure before metaphase (Gauthier et al., 1999; Fenech, 2000). A nuclear envelope is formed around such fragments or entire chromosomes in telophase and these structures serve as micronuclei. The development of centromere and kinetochore detection methods made it possible to distinguish the micronuclei formed as a result of chromosomal damage from those resulting from non-disjunction (Fenech, 1997). Thus, evaluation of the number of micronuclei in cells is a highly sensitive indicator of chromosome sensitivity. MN induction is commonly regarded as a biomarker of diseases and processes associated with genetic material damage (Cheng et al., 1996; Duffaud et al., 1997; Fenech, 2000; Umegaki and Fenech, 2000). The MN test allows for a reliable examination of chromosome loss or damage to an organism exposed ex vivo to genotoxic and cytotoxic agents (Fenech and Rinaldi, 1994; Fenech et al., 1999a, b).

The analysis of chromosomal instability is of the utmost importance for cytogenetic diagnosis as it evaluates DNA integrity in chromosomes and chromatin structure. The present study was aimed to determine if and how the presence of chromosomal polymorphism in blue and silver foxes influences chromosome complex stability in interspecific hybrids of these foxes.

## 2 Material and methods

The study was performed with 36 farmed foxes belonging to three breeds: blue foxes (*Alopex lagopus*), silver foxes (*Vulpes vulpes*) and interspecific hybrids (*Alopex lagopus* crossed with * Vulpes vulpes*). An experimental group of 12 animals (6 males and 6 females) was established for each species. The study used cells obtained from in vitro culture of peripheral whole blood lymphocytes, collected from *V. cephalica antebrachii*. The samples of whole peripheral blood was obtained during routine veterinary examinations. Cell cultures were grown under standard conditions (72 h, T 37.5 ${}^{\circ }$C, 5 % CO, with stable humidity). Cytochalasin B (5 µg mL${}^{-1}$) was added at 44 h. This material was used to prepare suspension preparations, which were subsequently stained by Giemsa (pH =6.8 in phosphate buffer). The preparations were stained and the damage identified based on the technique described by Słonina and Gasińska (1997). Acridine orange solution was used as an alternative to routine Giemsa staining in the MN test.

Microscopic analysis and micrographs were made in the Zeiss Imager A2 epifluorescence microscope fitted with a Zeiss AxioCam MRc5 camera. The test was evaluated with NIS-Elements ver. F2.31. In each preparation, 1000 binucleated cells (BNCs) that met the criteria of MN test evaluation were assessed (Fenech, 2000).

All analyses were performed using SAS software (SAS, 2014). Data (% MN and % BNC) were tested for normality before analysis using the Kolmogorov–Smirnov test included in PROC UNIVARIATE. Because % MN is a trait without normal distribution, the analysed trait was log transformed. After the transformation, distribution of the trait did not differ from normal. The tables present the results for % MN before transformation. The % BNC is a normally distributed trait. The PROC GLM (SAS, 2014) was used to perform analysis of variance, accounting, in the model, for animal species and sex analysed within animal species. Significant differences between the species were tested with the Tukey–Kramer test. Differences in the sex within species were analysed using Student's t test.

## 3 Results

**Figure 1:**
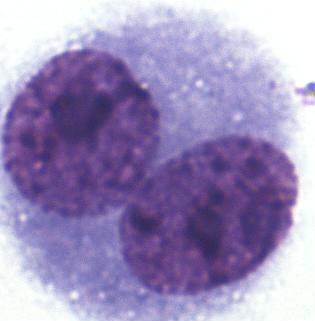
Binucleated cell (BNC) of blue fox; Giemsa staining.

To determine the degree of chromosome damage at the micronuclear level in somatic cells, about 220 000 cells in the interspecific hybrids, 55 910 cells in the blue foxes, and 35 455 cells in the silver foxes were counted. Multinucleate, necrotic and apoptotic cells were not analysed. BNCs for the MN test were selected based on the following traits: BNC, both nuclei with an intact nuclear envelope, both nuclei equal in size, both nuclei equal in staining pattern and staining intensity, both nuclei attached by a thin nucleoplasmic bridge – which is no wider than one-quarter of the nuclear diameter, and both nuclei touch but do not overlap. Cells with two overlapping nuclei were only accounted for when the boundaries of both nuclei were visible or cytoplasm boundary or nuclear envelope of BNCs were intact and distinguishable from the nuclear envelope of the adjacent cell. Damaged cells, overlapping cells and those with abnormal nuclei were also rejected. The micrograph is shown in Fig. 1.

**Figure 2:**
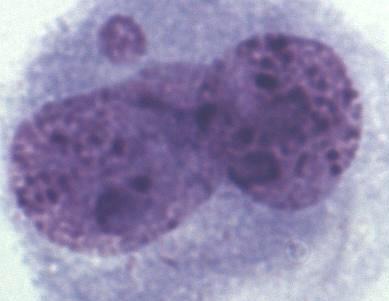
Binucleated cell (BNC) with one micronucleus (BNC + MN) in interspecific hybrids; Giemsa staining.

A total of 1000 BNCs were selected for each species. The micrograph from this part of the analyses is presented in Fig. 2. Highly significant differences between species were found for both % MN and % BNC (Table 1). All the three species differed in percentage of BNCs, with the lowest average observed in the interspecific hybrids (6.82) and highest average in silver foxes (35.86). The proportion of BNCs in the analysed biological material was estimated to be about 6 % in interspecific hybrids, 25 % in blue foxes, and 35 % in silver foxes. For the interspecific hybrids, the percentage of BNCs with micronuclei (BNC + MN) was about 15 %. The interspecific hybrids differed from the other fox species when % MN was analysed. The average value was lowest in blue foxes (3.33 %) and highest in interspecific hybrids (15.21 %). The lower BNC + MN values were estimated for two other species: about 3 % in blue foxes and about 4 % in silver foxes. The interspecific hybrids as an interspecies cross of polymorphic (B chromosomes – silver fox and Robertsonian translocation – blue fox) fox species are characterized by increased chromosomal instability, which may have a considerable influence on the health and breeding value of these animals.

In addition, differences in % BNC and in % BNC + MN were estimated between males and females, i.e. within sexes in individual species. For % BNC + MN, no significant differences were noted between the sexes within species (Table 2). The sex within fox species had a significant effect on percentage of binucleated cells. In the case of % BNC, the differences between sexes were observed for the blue foxes. Only interspecific hybrids shared a similar BNC value of about 6 %. These mean values differed for blue and silver foxes. Female blue foxes were characterized by considerably lower % BNC (17.67±5.31) compared to males (33.74±8.25). In the silver foxes, differences between females and males were small (35.26±7.57 vs. 36.46±8.88). The differences in % BNC were significant (P<0.05) only for the blue foxes.

**Table 1:** Percentages of BNC and BNC + MN

		% MN		% BNC	
Species	No. of	Average	SD	Average	SD
	animals				
Hybrid	12	15.21${}^{a}$	9.17	6.82${}^{a}$	3.03
Blue fox	12	3.33${}^{b}$	2.18	25.71${}^{b}$	10.68
Silver fox	12	3.92${}^{b}$	1.53	35.86${}^{c}$	7.89

${}^{a,\;b}$Means with different letters differ highly significantly (P<0.01).

Furthermore, differences in MN percent were estimated between males and females. The analysis of our results shows that % BNC + MN values were higher for females than for males in the case of the blue and silver fox species. For all species under analysis it was similar (about 4). For males it was lower at 2.67±1.08 for male blue foxes and 3.83±1.21 for male silver foxes. The difference between females and males was not significant for both species (P<0.05). The individual variation in % BNC + MN was different in the case of interspecific fox hybrids. % BNC + MN was lower in female hybrids (12.08±8.69) than in male hybrids (18.33±9.27), but the differences were not significant (P<0.05).

## 4 Discussion

Genomic instability might be observed as chromosomal instabilities and fragmentation of DNA strains in the cell nucleus by comet assay. Besides MN assay, chromosomal instabilities are evaluated by the sister chromatid exchange assay and the fragile site assay. Frequency of spontaneous sister chromatid exchange was analysed in various species of animals and amounted to 3.40 in chinchillas (Kuchta-Gładysz et al., 2015), 2.69 in rabbits and 1.41 in coypu (Kuchta-Gładysz et al., 2016), 3.38 in domestic cats (Szeleszczuk et al., 2014), 0.65 in blue fox and 2.33 in silver fox (Grzesiakowska et al., 2017). Cytogenetic instability identified as fragile site was observed in farm animals such as sheep (Wójcik et al., 2018) and in fur animals such as coypu (Kuchta-Gładysz et al., unpublished).

The analyses performed on farmed fox lymphocytes were aimed to evaluate chromosomal instability using the MN test. Cytokinesis-block MN assay involving cytochalasin B was used in the experiment. Cytochalasins are organic compounds that show pleiotropic effects on the cells and act on the actin cytoskeleton. Cytochalasin B is used to block cell division immediately after nuclear division. It blocks cytokinesis by inhibiting the polymerization of actin filaments and through fragmentation of microfilaments, without interfering with karyokinetic division (Flanagan and Lin, 1979). This results in the cells with two nuclei. Cytochalasin B is added to cell cultures before the first mitotic division, after inducing DNA damage. The cells that underwent complete nuclear division are next stored as BNCs (Fenech, 1997). The invention of the cytochalasin B technique allows for distinguishing the cells which did not undergo nuclear division from those that completed this division. Thus, it compares the extent of genetic material damage between populations of cells differing in division kinetics. This discovery represented a significant improvement in many fields of science, including cell biology and toxicology (Fenech, 2000). In our study we used cytochalasin B at a dose of 5 µg mL${}^{-1}$ and obtained 20 % normal binucleated cells on average. This means that every fifth cell in the preparation met the criteria of MN test evaluation. It appears that the dose of 5 µg mL${}^{-1}$ cytochalasin B should be sufficient to block cytokinesis. Fenech (2000) used 4.5 and 6 µg mL${}^{-1}$ cytochalasin B. However, these doses were proposed and used for human cell lines. In animals, a dose of 2 µg mL${}^{-1}$ was used in fish (Çavaş and Ergene-Gözükara, 2005), 3 µg mL${}^{-1}$ in hamster (Matsushima et al., 1999), and 5 µg mL${}^{-1}$ in mice (Ren et al., 1991).

**Table 2:** Comparison of males and females in the MN test

			% BNC + MN		% BNC	
Species	Sex	No. of	Average	SD	Average	SD
		animals				
Hybrid	F	6	12.08${}^{a}$	8.69	6.74${}^{a}$	3.00
	M	6	18.33${}^{a}$	9.27	6.90${}^{a}$	3.36
Blue fox	F	6	4.00${}^{a}$	2.86	17.67${}^{a}$	5.31
	M	6	2.67${}^{a}$	1.08	33.74${}^{b}$	8.25
Silver fox	F	6	4.00${}^{a}$	1.92	35.26${}^{a}$	7.57
	M	6	3.83${}^{a}$	1.21	36.46${}^{a}$	8.88

${}^{a,\;b}$ Means within a species denoted with different letters differ significantly (P<0.05).

Chromosomal instability was evaluated based on frequency of micronuclei in somatic cells of the foxes. The higher the MN percentage in an individual, the more unstable the genetic material in the cells. Special care should be exercised when identifying the micronuclei. Staining artifacts may often interfere with the evaluation of the preparation (Al-Sabti and Metcalfe, 1995). Therefore, specific criteria have been established to analyse the micronuclei themselves (Fenech, 2000). The diameter of the MN in human lymphocytes varies between 1/16 and 1/3 of the mean diameter of the main nucleus, which corresponds to between 1/256 and 1/9 of the diameter of one of the main nucleus in a BNC. The MN may touch but not overlap the main nucleus and the micronuclear boundary should be distinguishable from the nuclear boundary.

The MN has the same staining intensity as the main nucleus but occasionally staining may be more intense (Fenech, 2000). Another questionable step in the MN test procedure is the number of BNCs subjected to statistical analysis. In our study, we counted and selected BNCs per 1000 proper binucleated cells. In a study with fish (Metcalfe, 1988), the minimum number of counted binucleated erythrocytes was assumed to be 4000. Flow cytometry allows for counting a much greater number of cells: such as 20 000 cells (Kissling et al., 2007) or over 50 000 cells (Schultz et al., 1993). Al-Sabti (1994) demonstrated that it is sufficient to analyse 500 binucleated cells. Çavaş and Ergene-Gözükara (2005) analysed 1500 cells. In a study with mammals, birds and reptiles, Zúñiga-González et al. (2000) counted 10 000 cells per animal.

Initially, the MN technique was only used to detect chromosome damage or loss. Recently it has been modified in many ways to obtain information about the frequency of binucleated cells in culture as an indicator of the mitotic index; apoptosis in mononuclear or binucleated cells as an indicator of cell death before or after cell division; and discrimination of aneugenic and clastogenic changes through the combination of the FISH technique for centromeres or telomeres with MN technique. It also allows for detection of specific sequences in both the micro- and macronucleus, thus identifying abnormal chromosome number in the cell caused by no pairing of homologous chromosomes or chromosome loss (Kirsch-Volders et al., 1997). This technique is also used to determine the number of apoptotic or necrotic cells based on special criteria that distinguish these cells from live cells, taking into account their morphology (Fenech, 2000). The assay is also applied to test the degree of genetic material damage in cells exposed to gamma radiation (Chang et al., 1997), butadiene (Tates et al., 1996), benzene, clastogenic ionizing radiation, and aneuploidogens such as the cytostatic drug vinblastine, phenylalanine, thiotepa, busulfan, methotrexate (Norppa et al., 1993; Anwar et al., 1994) or tobacco smoke (Tomanin et al., 1991). It is also possible to test the individual immunity of an organism exposed to certain substances or environmental conditions. There are a number of relationships between MN frequency and factors such as species, sex, reproductive status, age, health status, nutrition, and even vitamin content (Fenech and Rinaldi, 1994, 1995; Fenech, 1998; Fenech and Crott, 2002). Furthermore, studies are being carried out into the effect of nutrition on the MN test results. One of them determined the number of micronuclei in lymphocytes in persons on a conventional diet and in vegetarians. The MN index was much lower in meat-eating men than in vegetarians (Fenech and Rinaldi, 1995). High plasma levels of folic acid and vitamin B${}_{12}$ were also found to reduce the number of micronuclei in lymphocytes (Fenech and Rinaldi, 1994). This dose was considerably higher than the average plasma content of folic acid (<30 nM; Fenech and Crott, 2002). Fenech et al. (1999a) investigated cytotoxic and genotoxic effects of hydrogen peroxide on human lymphocytes. Increased concentration of hydrogen peroxide was found to be associated with increased MN frequency. Çelik et al. (2003) studied the effect of long-term exposure to benzene in petrol station attendants. To this end, the number of micronuclei in buccal epithelial cells was analysed. The number of micronuclei was considerably higher among the workers than in the control group and was correlated to the tobacco smoking habit. The same study determined the frequencies (‰) of micronuclei for the analysed group to be 1.63±0.08 in smoker attendants and 1.14±0.06 in non-smoker attendants, compared to 0.65±0.03 in control smokers and 0.29±0.02 in control non-smokers. Higher levels of chromosome damage were also observed both in the attendants and in tobacco addicts from the control group. The MN test performed with epithelial cells is a useful biomarker to assess occupational exposure to genotoxic chemical substances. However, according to some scientists, lymphocytes are considered the best material to test DNA damage, because they circulate through all tissues and organs of the body, thus providing reliable information concerning the effect of mutagens on the cells (Tucker and Preston, 1996). For this reason, lymphocytes from whole peripheral blood were used in our study. The MN assay was performed to assess chromosomal instability in animals, hybrids of two polymorphic species: the blue fox and the silver fox.

Zúñiga-González et al. (2000) studied the number of spontaneous nuclei in different species of mammals, birds and reptiles. The animals with the highest frequency of micronuclei were ocelot – 13.5 MN per 10 000 cells; lynx – 10.8; owl – 10.6; gray squirrel – 9.1; hedgehog – 9.2; lion – 4.1; orange-fronted parakeet – 5.1; and common barn owl – 3.5. The lowest number of spontaneous micronuclei was observed in animals such as coati – 0.1; raccoon – 0.1; mouflon sheep – 0.3; and American crocodile – 1.2. Black bear, coyote, alligator snapping turtle and black-crowned night heron had a MN content equal to zero. Gauthier et al. (1999) compared the degree of genetic material damage among marine mammals and the number of micronuclei ranged from 2 to 14 per 1000 binucleated cells. The MN test also found application in Canidae species. Harper et al. (2007) investigated the effect of the cytotoxic drug cyclophosphamide on reticulocytes of beagle dogs and reported the number of micronuclei to be around 8. In our study, MN number in the analysed foxes was 15 in interspecific hybrids, 3 in blue foxes, and about 4 in silver foxes.

A very interesting experiment was carried out by Ramírez-Muñoz et al. (1999) who investigated the number of micronuclei in animals with spleen and in animals without this organ. The aim of the observations was to determine if the spleen of species such as hamster, rat, mouse, rabbit and dog is capable of removing erythrocytes with micronuclei. Indeed, splenectomized animals had a higher number of micronuclei in the cells (mouse: 34.8; rat: 3.1; dog: 12.7 MN per 10 000 cells) compared to non-splenectomized animals (mouse: 28; rat: 2.4; dog: 1.1 MN).

The main advantage of the MN test is that it allows for easy counting of micronuclei and for analysing a significantly greater number of cells than during observation of metaphase plates. Classic cytogenetic techniques examine chromosomes directly by analysing metaphase plates, which delivers more detailed information but is more complex and labour consuming (Fenech, 2000). The MN technique is a relatively simple analysis that provides adequate information about chromosome damage and loss but fails to detect subtle changes such as balanced translocation. Cytokinesis-block MN assay has the important advantage of reliable identification of the cells that underwent a single, complete nuclear division; another advantage is its sensitivity and precision. However, the MN test is not without flaws. Fenech (1997) suggested that certain micronuclei can already be present in lymphocytes prior to culture rather than being formed as a result of DNA damage of centromere and kinetochore defects. Lymphocytes may include micronuclei formed in vivo during the last cell division prior to culture (Castelain et al., 1993).

In our study, the MN test was used to estimate the effect of crossing highly polymorphic species on their genetic material. It is concluded from our results that the widespread chromosome polymorphism in interspecific hybrids does influence their somatic cell instability. The level of chromosome damage in the hybrid lymphocytes was considerably higher than in the cells of blue and silver foxes. This is due to the fact that the karyotype of interspecific hybrids results from fusion of unstable karyotypes from both parents. The karyotype polymorphism present in the cells of blue foxes is the outcome of a Robertsonian translocation. This balanced mutation involves a shift of a chromosome fragment between two or more chromosomes, without a loss or increase of genetic material in the cell (Świtoński et al., 2006). This translocation spreads easily throughout the population due to its simple inheritance. It occurs in many species such as cattle, pigs, goats, sheep and dogs (Świtoński et al., 2006). The unstable karyotype present in silver foxes is due to the occurrence of accessory B chromosomes in the cells. Supernumerary chromosomes were also detected in some species of marsupials, bats, primates, rodents and even-toed ungulates (Volobujev, 1981). B chromosomes differ among species in morphology and certain characteristics. Three types of B chromosomes are distinguished; in foxes these are microchromosomes (Volobujev, 1981; Świtoński et al., 2006; Bugno-Poniewierska et al., 2013). To obtain more information about the instability of genetic material in the cells of these animals, studies should be extended with other cytogenetic tests to complement the present research. It is also necessary to perform studies on animals originated from other farms.

The chromosome polymorphism found in blue and silver foxes, resulting from a variable number of chromosomes, had little effect on genetic material stability in the somatic cells. The unstable karyotype of interspecific hybrids significantly increases the incidence of chromosomal damage, as manifested by the increased frequency of micronuclei in the cells.

## Data Availability

No data sets were used in this article.
